# Lack of XBP-1 Impedes Murine Cytomegalovirus Gene Expression

**DOI:** 10.1371/journal.pone.0110942

**Published:** 2014-10-21

**Authors:** Adi Drori, Martin Messerle, Wolfram Brune, Boaz Tirosh

**Affiliations:** 1 Institute for Drug Research, The Hebrew University of Jerusalem, Jerusalem, Israel; 2 Department of Virology, Hannover Medical School, Hannover, Germany; 3 Heinrich Pette Institute, Leibniz Institute for Experimental Virology, Hamburg, Germany; University of Regensburg, Germany

## Abstract

The unfolded protein response (UPR) is an endoplasmic reticulum (ER)-to-nucleus signaling cascade induced in response to ER stress. The UPR aims at restoring homeostasis, but can also induce apoptosis if stress persists. Infection by human and murine cytomegaloviruses (CMVs) provokes ER stress and induces the UPR. However, both CMVs manipulate the UPR to promote its prosurvival activity and delay apoptosis. The underlying mechanisms remain largely unknown. Recently, we demonstrated that MCMV and HCMV encode a late protein to target IRE1 for degradation. However, the importance of its downstream effector, X Box binding protein 1 (XBP-1), has not been directly studied. Here we show that deletion of XBP-1 prior to or early after infection confers a transient delay in viral propagation in fibroblasts that can be overcome by increasing the viral dose. A similar phenotype was demonstrated in peritoneal macrophages. In vivo, acute infection by MCMV is reduced in the absence of XBP-1. Our data indicate that removal of XBP-1 confers a kinetic delay in early stages of MCMV infection and suggest that the late targeting of IRE1 is aimed at inhibiting activities other than the splicing of XBP-1 mRNA.

## Introduction

Cytomegalovirus (CMV) is the prototype member of the β-herpesvirus subfamily (β-Herpesvirinae), harboring a linear double stranded DNA genome. Human cytomegalovirus (HCMV) infects 50%–90% of populations worldwide and is the most ubiquitous infectious pathogen at any age. Despite the high prevalence, CMV infection of immune competent individuals is usually asymptomatic. However, human cytomegalovirus is a leading cause of severe morbidity and mortality in immunocompromised individuals, including AIDS patients, organ transplant recipients, and congenitally infected newborns [1], [2]. Due to its strict species specificity, the study of HCMV pathogenesis cannot be investigated in animal models and thus is limited to clinical samples and human cell lines. Therefore, rodent cytomegaloviruses have been used to address mechanistic questions regarding replication and pathogenesis. The most extensively used model is the murine cytomegalovirus (MCMV), which presents a remarkable resemblance to HCMV with respect to pathogenesis during acute infection, establishment of latency and reactivation and the induction of immune responses. Importantly, MCMV shares HCMV genome size of about 230 kb, the sequential gene expression pattern and the tropism for hematopoietic cells and cells of secretory glands [3]–[7].

The large genome of both viruses potentially encodes up to 200 proteins. Many of them are abundantly expressed and are destined to enter the endoplasmic reticulum (ER), where they acquire a folded state and undergo various post-translational modifications. As a consequence, a productive viral infection loads the ER with copious amount of proteins that inevitably exceed the organelle's folding capacity, leading to conditions of ER stress [8], [9]. To counteract ER stress, eukaryotic cells evolved ER-to-nucleus signaling cascade collectively referred to as the unfolded protein response (UPR). UPR attempts to restore homoeostasis by increasing the folding and secretory capacity of the ER and simultaneously diminish global protein translation [10], [11]. The canonical mammalian UPR operates via three independent branches named by the three ER resident sensors PKR-like ER kinase (PERK), activating transcription factor 6 (ATF6) and inositol-requiring enzyme 1 (IRE1). When activated, PERK is autophosphorylated within seconds and then phosphorylates the translation initiation factor eIF2α, leading to dramatic attenuation of global protein synthesis, alongside increased translation of the transcription factor ATF4. ATF4 initiates a negative feedback loop to allow resumed translation, favors the translation of redox genes, such as ER oxidoreductin 1 (ERO1), and promotes autophagy [12]. ATF6 is activated within minutes to hours after infliction of ER stress. Upon activation ATF6 leaves the ER to enter the Golgi, where it undergoes intramembranous proteolysis. The cleaved protein then travels to the nucleus where it activates the transcription of chaperone genes (e.g. binding immunoglobulin protein,BiP), genes of the ER associated degradation (ERAD) pathway as well as of the gene encoding the X-box binding protein 1 (XBP-1) [13], [14]. In parallel, IRE1 is activated by trans-autophosphorylation allowing the activation of its endonuclease domain, which cleaves the mRNA of XBP-1 to yield the spliced form of XBP-1 (XBP-1s). This mRNA gives rise to a 371 amino acids transcription factor that comprises a DNA binding domain plus a potent transactivation domain. XBP-1s promotes the expression of a large number of genes, whose products facilitate ER biogenesis, protein folding in the ER, and degradation of terminally misfolded proteins [13], [15]. Thus, XBP-1 is a key factor in alleviating ER stress by increasing ER folding capacity on one hand, and curtailing the hazardous effect of accumulating unfolded and misfolded proteins on the other. However, prolonged ER stress leading to sustained UPR activation that fails to relief the burden of unfolded proteins will ultimately cause cell cycle arrest and initiate cell death [16], [17]. Accumulating evidence indicates that the mechanism of ER stress-mediated apoptosis is controlled by the UPR itself, primarily the PERK and IRE1 pathways. IRE1, under unabated ER stress, promotes activation of the JNK pathway [18]. In addition, the nuclease activity of IRE1 is diverted from XBP-1 mRNA splicing towards mRNA and miRNA degradation, which attenuate the expression of prosurvival proteins and enhance the expression of proapoptotic proteins [19]. This non-XBP-1 splicing nuclease activity is referred to as regulated IRE1-dependent decay (RIDD) and is accentuated in the absence of XBP-1 [20], [21].

Moreover, genetic studies demonstrated diverse roles of UPR key components that are not directly linked to ER function and protein folding. For instance, XBP-1s promotes the expression of the inflammatory cytokines IL-6 and TNFα as well as interferon-β following induction of TLR2 and TLR4 in macrophages [22]–[24]. XBP-1 splicing was also shown to be induced by the p38-MAPK pathway in *C. elegans*, and conferred larval development and survival following bacterial infection [25]. A role in protection of the gut mucosa from invasion of bacteria was also demonstrated [26], as well as regulation of lipid and glucose metabolism [27]. In addition, the UPR plays developmental roles for various cell types with a high secretory capacity [28]–[30]. These processes affected by the UPR clearly have the potential to influence viral replication in host tissue. Some processes may be beneficial for viral propagation, whereas others might serve the host to restrict it. Therefore, it is not surprising that viruses have evolved means to actively interfere with UPR signaling to their own benefit by multiple mechanisms. Indeed, the manipulation of UPR by CMV is a conserved strategy and was demonstrated in both HCMV and MCMV. Both HCMV and MCMV target PERK-ATF4 and IRE1-XBP-1 pathways to selectively activate a subset of UPR genes [31]. Interestingly, although XBP-1 mRNA was spliced upon infection with HCMV, its target genes involved in protein degradation were not expressed. Moreover, ATF6 cleavage was inhibited in infected cells, but chaperone genes were expressed in an ATF6 independent manner [9]. HCMV also harnesses the PERK pathway of the UPR to manipulate cellular metabolism for its benefit [32].

Recently we identified the late proteins M50 and UL50 encoded by MCMV and HCMV, respectively, to interact with IRE1. This interaction leads to IRE1 degradation [33]. Owing to the multifaceted roles played by IRE1 following activation, which include the splicing of XBP-1 mRNA, RIDD, JNK activation and triggering innate immunity sensors [34], it is not clear which of all of these have the potential to affect viral propagation in infected cells. This can be address by investigating the specific roles of the elements downstream to IRE1. Here we rigorously explored the role of XBP-1 in MCMV infection in vitro and in vivo.

## Materials and Methods

Unless stated otherwise, all materials were purchased from Sigma (St. Louis, MO, USA). Solvents were of analytical grade or higher. Water was deionized and ultra-filtered by reverse osmosis (Barnstead Nanopure, Waltham, MA).

### Ethics statement

All animal studies were conducted in accord with the Principles of Laboratory Animal Care (NIH publication #85-23, revised1985) under protocol no. MD-13071. The joint ethics committee (IACUC) of the Hebrew University and Hadassah Medical Center approved the study protocol for animal welfare. The Hebrew University Animal Facility is an AAALAC international accredited institute (#1285).

### Mice

Mice containing a conditional floxed allele of XBP-1 (XBP-1^f/f^, described in [34]) were crossed to mice knocked in for a Cre-estrogen receptor fusion protein into their RNA polymerase heavy subunit (termed RERT). These mice were kindly provided by Dr. Mariano Barbacid (Spanish National Cancer Research Centre, Madrid, Spain) [35]. The crossed homozygous mice are described here as RERT/XBP-1^f/f^. WT C57BL/6 mice were purchased from Harlan Israel. All mice were housed in indicidual ventilated cages and maintained under specific-pathogen-free conditions. Male mice were used at 8–10 weeks for all experiment, excluding MEFs isolation.

### Isolation of Peritoneal Macrophages (PMs)

Primary Peritoneal Macrophages were isolated from male mice, injected i.p. with 2.5 ml of aged thioglycolate broth (3% w/v, Becton Dickinson, Franklin Lakes, NJ, USA). 4 days post injection mice were sacrificied and PMs were isolated by peritoneal lavage with 5 ml of PBS. Erythrocytes were eliminated by 3 min incubation of isolated cells in ACK buffer (0.15 M NH_4_Cl, 10 mM KHCO_3_, 0.1 mM EDTA, pH = 7.4).

### Cell culture

Mouse embryonic fibroblasts (MEFs) were grown in Dulbecco's modified Eagle's medium (DMEM) (Biological Industries Beit Haemek, Israel) supplemented with 10% fetal calf serum, 2 mM glutamine, 100 unit/ml penicillin, and 100 µg/ml streptomycin. 0.25% Trypsin solution was used for harvest. PMs were grown in RPMI medium supplemented with 10% fetal calf serum, 2 mM glutamine, 100 unit/ml penicillin, 100 µg/ml streptomycin, 10 mM HEPES (pH = 7.4), 1% non-essential amino acids, 1 mM sodium pyruvate and 50 µM β-mercaptoethanol. Accutase cell detachment medium (eBiosciences, San Diego, CA) was used to harvest PMs. Cells were maintained at 37°C in a 5% CO_2_ incubator.

### Viruses and infection of cells and mice

Viruses used in this study were derived from the pSM3fr BAC plasmid, containing the whole Smith strain MCMV genome [36]. WT virus is designated here as MCMV, and recombinants expressing GFP [37], Cre recombinase [38] and firefly luciferase [39] are designated as MCMV-GFP, MCMV-CRE and Luc-MCMV, respectively. All viruses were reconstituted and tittered as described before [40].

Viruses were diluted in growth medium and used to infect cultured cells at MOI of 0.1 or 1 pfu/cell, as indicated. For in vivo infection of mice, viral stocks were diluted to the indicated concentration in PBS and 200 µl of the solution was injected to tail vein.

### BAC mutagenesis

WT-MCMV-Δm157-EGFP and MCMV-CRE-Δm157-EGFP genomes were generated by introducing the GFP ORF under control of the Ubiquitin C promoter (UbC) into the m157 locus of MCMV or MCMV-CRE applying homologous recombination-based mutagenesis in E. coli as described [41]. In brief, the UbC promoter and the GFP ORF were cloned next to a kanamycin resistance (kanR) gene within plasmid pOri6K-F5. The whole cassette was then PCR amplified using the primers 5′-CCCGGGGCCTTCACGGTAAGGATCTGACAGTCGACCGTCGATTCGTCAGTATAGATCTATCCAGTTTGGACT-3′ and 5′-AATCTGAACCCCGATATTTGAGAAAGTGTACCCCGATATTCAGTACCTCTTCAGGAACACTTAACGGCTG-3′, and the resulting PCR product was electroporated to WT-MCMV or MCMV-CRE BAC-containing E.coli. Following selection of bacterial clones with kanamycin and chloramphenicol recombinant BACs were isolated and characterized by restriction analysis. The KanR cassette was subsequently excised by Flp recombinase, and viral mutants were reconstituted by transfection of murine fibroblasts with BAC DNA.

### Induction of XBP-1 deletion

#### In-vivo

Male RERT/XBP-1^f/f^ mice were injected with a series of three subcutaneous injections of 100 µl tamoxifen dissolved in corn oil (10 mg/ml). RERT/XBP-1^WT/WT^ control mice were injected with same volumes of vehicle.

#### In-vitro

Cultured cells were treated with a final concentration of 2.5 µM (MEFs) or 5 µM (peritoneal macrophages) of 4-hydroxytamoxifen (4-OHT) dissolved in ethanol (EtOH) for the indicated time before viral infection.

DNA was extracted from cells or tissue using KAPA Mouse Genotyping Kit (Kapa Biosystems, Wilmington, MA) and was used as template in PCR reaction with primers INT1-S: 5′ CTTTGTGGTCGTAGGGTAGGAACC-3′, lox-S: 5′-ACTTGCACCAACACTTGCCATTTC-3′ and lox-A: 5′-CAAGGTGGTTCACTGCCTGTAA TG-3′. PCR products were resolved on 2.5% agarose gel to separate WT (183 bp) and deleted (352 bp) alleles of XBP-1.

### Flow Cytometry

Mock cells or cells infected with GFP-expressing viruses were harvested at the indicated time points postinfection and fixed in PBS containing 0.1% paraformaldehyde. Samples from one time-course experiment were analyzed simultaneously. GFP-expressing cells were gated and counted as infected cells.

Single cell suspensions of PMs were stained with anti-mouse F4/80-FITC (clone BM8 from Biolegends) for 30 min at 4°C. Mouse IgG2A serves as isotype control to set gate parameters. Cell viability was assessed using staining with 5 µl of 10 µg/mL propidium iodide in PBS. All samples were analyzed using a BD™ LSR II flow cytometer (Becton Dickinson, Franklin Lakes, NJ) and collected data was analyzed by FCS Express V3 (De Novo software, CA, USA).

### Immunoblotting

Cell lysates were prepared in 100 µl of RIPA buffer (25 mM Tris•HCl pH 7.6, 150 mM NaCl, 1% NP-40, 1% sodium deoxycholate, 0.1% SDS) by vigorous vortex and shaking. Protein concentration was measured using a BCA kit (Pierce). Samples were resolved by SDS-PAGE (12% acrylamide) and transferred to PVDF membranes. Blots were incubated with primary antibodies over night at 4°C. α-mouse or α-rabbit HRP-conjugated secondary antibodies were used followed by chemiluminescence detection. Monoclonal antibodies against MCMV IE1 and E1 (hybridoma supernatants) were kindly provided by Prof. Stipan Jonjic (Department of Histology and Embryology, Faculty of Medicine, University of Rijeka, Croatia). For late phase protein detection the monoclonal antibody 20/352/4 was used, which recognizes a yet uncharacterized protein.

### Isolation of nuclear DNA

Infected MEFs were harvested using 0.25% Trypsin solution, washed twice with ice-cold PBS and ressuspended in 200 µl of Hypotonic buffer (20 mM Tris-HCl (pH 7.4), 10 mM NaCl, 3 mM MgCl2). After 15 min incubation samples were centrifuged, and DNA was isollated from the pellets using DNeasy Blood & Tissue kit (Qiagen).

### Quantification of viral genome copy number

Total DNA or nuclear DNA was purified from mock-infected or MCMV-infected cells. The levels of the viral gene M55(gB) and the host gene pThrp were measured by quantitative PCR analysis. pDrive-gB-PTHrP plasmid was used as a template to create a calibration curve, and actual genome copy numbers were calculated from qPCR data and calibration curves.

### Quantitative Real Time PCR

Total RNA was isolated using RNeasy Plus Mini kit (Qiagen). One microgram of total RNA was transcribed into cDNA using RevertAid first strand cDNA synthesis kit with random hexameric primers, according to manufacturer's instructions (Fermentas). Real-time PCR reactions were performed using KAPA SYBR FAST Universal Mix (Kapa Biosystems) and CFX connect real-time system (Bio-Rad). Ubiquitin C mRNA was used as normalizer in all experiments unless otherwise indicated. The following primers were used for qPCR analysis of MCMV genes (adopted from [31]) IE1 forward: 5′-CAGGGTGGATCATGAAG CCT-3′, IE1 reverse: 5′-AGCGCATCGAAAGACAACG-3′, EI forward: 5′-GAATCCGAGGA GGAAGACGAT-3′, EI reverse: 5′-GGTGAACGTTTGCTCGATCTC-3′, M55(gB) forward: 5′-GCGATGTCCGAGTGTGTCAAG-3′, M55 (gB) reverse: 5′-CGACCAGCGGTCTCGAATA AC-3′.

### Radioactive pulse-labeling

Pulse labeling was performed as follow: after starvation in methionine/cysteine-free Dulbecco's modified Eagle's medium for 45 min, equal numbers of cells were metabolically labeled at 37°C for 10 min with [^35^S]methionine/cysteine (7.5 µCi/500 µL) (Perkin Elmer, USA). Cells were then lysed in lysis buffer (50 mM Tris pH = 8, 200 mM NaCl, 20 mM MgCl_2_,1% NP-40, 3 µL/mL normal rabbit serum, 10 µL/mL BSA and 0.1% protease inhibitors), containing 1% SDS. Lysates were resolved by SDS-PAGE under reducing conditions and visualized by fluorography.

### Immunohistochemistry

Immunohistochemical analysis was performed on 10 µm sections from formalin-fixed, paraffin-embedded livers. The same monoclonal antibodies used for Western blot analysis was used to stain tissue sections. Briefly, a 20 min heat-induced epitope retrieval (10 mM citrate buffer. pH 6.0) was followed by blocking for 10 min with 3% H_2_0_2_ in methanol. Incubation with the primary antibody (1∶100) was then performed overnight at 4°C. This was followed by incubation with horse anti-mouse HRP-conjugated for 30 min at room temperature. Signal was enhanced using VECTASTAIN Elite ABC Kit (Vector Laboratories). Staining was complete with DAB Peroxidase (HRP) Substrate kit (SK-4100, Vector Laboratories) for 10 min followed by counterstaining for 30 seconds with hematoxylin.

## Results

### Impaired growth and impeded protein expression of MCMV in XBP-1 KO MEFs

**Figure 1:**
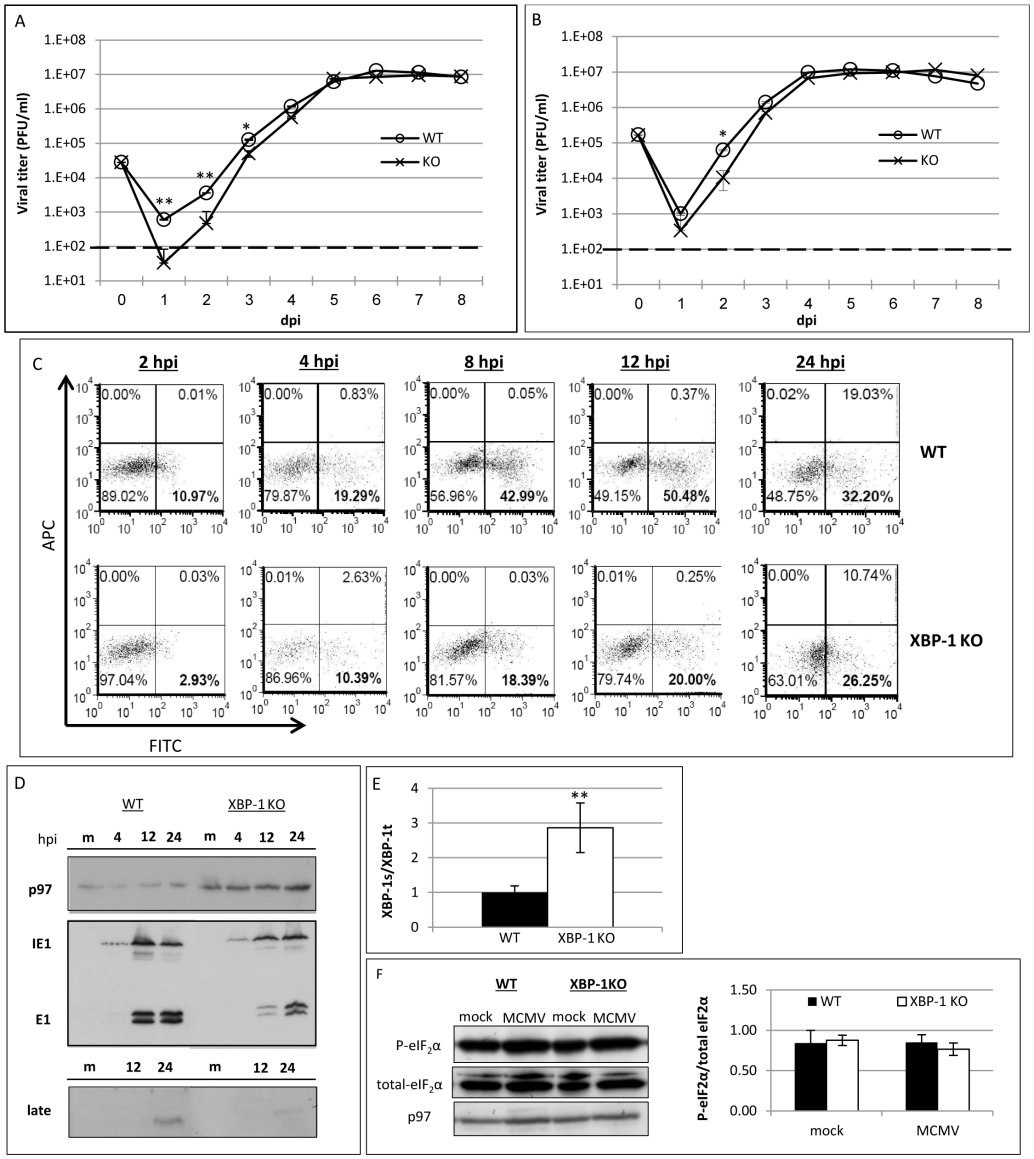
Delayed kinetics of MCMV protein expression in XBP-1 KO MEFs. Wild type or XBP-1 KO MEFs were infected with MCMV at MOI of 0.1 (A) or 1 (B). Culture supernatants were collected at indicated time points and titers were determined using standard plaque assay. (C) Flow cytometry analysis of MEFs, infected with MCMV-GFP at MOI = 1. GFP positive cells were gated and counted as infected cells. (D) The expression of intrinsic viral proteins was measured by Western blot analysis, using monoclonal antibodies against Immediate Early 1 (IE1), Early 1 (E1) and a 48 kDa late protein. Cellular p97 served as loading control. (E) mRNA was extracted from mock infected MEFs, and the ratio XBP-1s to total XBP-1 mRNAs was calculated from quantitative RT-PCR data. (F) Western blot analysis of phosphorylated and total eIF2α of mock and MCMV infected WT or XBP-1 KO MEFs. Blot was quantified using ImageJ and the ratio between phosphorylated and total eIF2α was calculated. Presented data is from one experiment out of three preformed (C, D, F). Results are shown as mean ±SEM of at least three replicates. * P<0.05, ** P<0.01.

To address the role of XBP-1 in MCMV acute infection we used immortalized MEFs derived from WT or XBP-1 KO mice. Cells were infected with BAC-derived MCMV at an MOI of 0.1 and viral progeny titers were analyzed up to 8 days post infection. Titers derived from XBP-1 KO cells decreased substantially compared to those derived from WT cells at 1 day post infection (dpi) (Fig. 1A). Notably, this difference gradually diminished throughout the infection until it was no longer apparent at 5 dpi. When the same cells were infected at an MOI of 1 (Fig. 1B), we observed milder differences at day 1 and viral titers from XBP-1 cells reached WT levels by day 4 post infection. These data suggest that XBP-1 contributes to viral propagation in the early stages of infection in an MOI-dependent manner.

The differences in growth at the early time-points prompted us to analyze the early stages of the first infection cycle more closely. To do so, we used EGFP-expressing viruses in which the GFP gene is driven from the heterologous MIE promoter of HCMV inserted into the immediate early 2 locus (MCMV-GFP). Cells were infected with the MCMV-GFP virus at an MOI of 1 and the number of infected cells during the first 24 h post infection was measured by flow cytometry. We chose MOI of 1 over 0.1 despite the lower differences in viral titers, since it offered a better detection of viral RNA and protein during early times of infection. It should be noted that as the infection proceeded beyond the first 24 h, the number of GFP-positive cells sometimes diminished most likely owing to cell lysis. Moreover, at this time point viral progeny are released from the lysed cells and the number of GFP-expressing cells can no longer be compared between cultures. We therefore terminated the experiment at 24 h or when percentage of GFP positive cells started to decrease. The percentage of cells positive for the GFP reporter was lower in XBP-1 KO MEFs compared to WT, from 2 to 24 hpi, suggesting delayed kinetics of virally-derived protein expression in the absence of XBP-1 (Fig. 1C).

Because GFP is expressed from the IE2 locus, we decided to verify that XBP-1 also affects the expression of intrinsic viral proteins from all phases of the MCMV replication cycle. To this end, the expression of three proteins representative of the three phases of the infection cycle was assessed by immunoblotting: Immediate Early 1 (IE1), Early 1 (E1), and an as-yet uncharacterized late protein of 48 kDa detected by the monoclonal antibody 20/352/4. The expression of all three intrinsic viral proteins was reduced in XBP-1 KO cells compared to WT, indicating that the reduced GFP expression in XBP-1 KO reflects a general reduction in viral protein synthesis (Fig. 1D) and was consistent with the retarded generation of infectious progeny in XBP-1-deficient fibroblasts.

Owing to its major role in alleviating ER stress, the deletion of XBP-1 results in the chronic activation of ER stress conditions [42]. This stress is demonstrated by the elevated levels of XBP-1 splicing in KO cells (Fig. 1E). Chronic ER stress has been shown to affect many aspects of cell physiology that may impinge directly or indirectly on MCMV proliferation. Since total protein synthesis attenuation is a major characteristic of ER stress, we decided to check whether cellular protein translation is reduced in cells deleted for XBP-1. Therefore, we examined the level of eIF2α phosphorylation. We found that the ratio between phosphorylated and total eIF2α was comparable between WT and XBP-1 KO cells, suggesting that global protein synthesis is not attenuated in the KO cells, probably due to adaptation to the chronic ER stress conditions (Fig. 1F). However, in addition to protein synthesis, chronic ER stress has been shown by ourselves and others to affect metabolism, cell cycle and other biological variables relevant to viral protein expression [43], [44]. Furthermore, the immortalized MEFs have been passaged numerous times, and the differences in MCMV infectivity might be connected to changes that the cells endured during culture, perhaps unrelated to the presence or absence of XBP-1. To obviate these experimental caveats and to clearly establish a role for XBP-1 in MCMV infection we utilized an inducible XBP-1 KO system.

### Induction of XBP-1 deletion prior to infection impairs MCMV growth and decelerates viral protein expression

**Figure 2:**
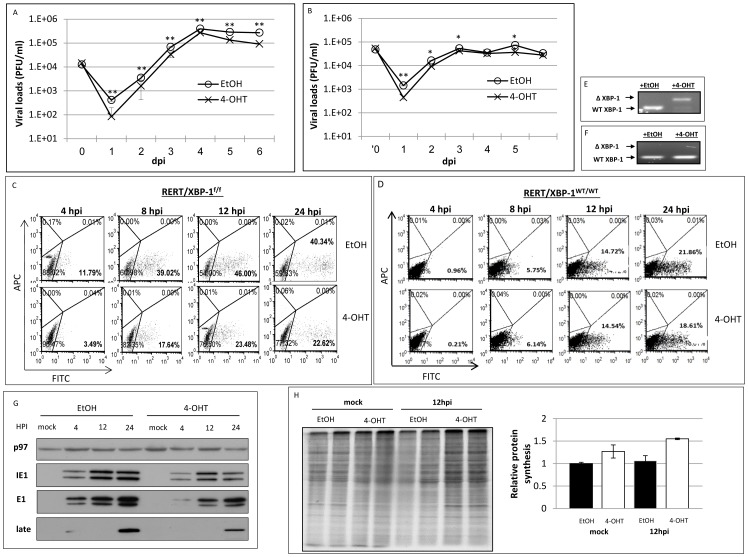
Induction of XBP-1 KO in primary MEFs prior to MCMV infection decelerates viral growth and protein expression. Primary MEFs derived from RERT/XBP-1^f/f^ mice were treated with vehicle (EtOH) or 4-OHT for 48 h and infected with MCMV-GFP atMO1 = 0.1 (A) and MOI = 1 (B). Culture supernatants were collected at indicated time points and titers were determined using standard plaque assay. (C) The expression of virus-derived GFP in RERT/XBP-1^f/f^ (C) and RERT/XBP-1^WT/WT^ (D) was monitored using flow cytometry. GFP positive cells were gated and considered as infected cells. (E, F) DNA was extracted from described cells and the genomic deletion of XBP-1 following EtOH or 4-OHT treatment was demonstrated using PCR analysis. Upper band represents deleted allele. (G) The expression of intrinsic viral proteins Immediate Early 1 (IE1), Early 1 (E1) and a 48 kDa late protein was measured by Western blot analysis. Cellular p97 served as loading control. (H) Total protein synthesis was assessed by radioactive pulse labeling and quantified using ImageJ. Data is from one experiment out of two (D, H), or three performed (C, G). (A, B) Results are shown as mean ±SEM of at least three replicates. * P<0.05, ** P<0.01.

We generated RERT/XBP-1^f/f^ mice that harbor a ubiquitously expressed Cre-ER fusion, thus when exposed to tamoxifen or its water soluble analog 4-hydroxytamoxifen (4-OHT), the XBP-1 allele undergoes Cre-mediated deletion [45]. MEFs were generated from the RERT/XBP-1^f/f^ mice and cultured for 48 h with 4-OHT. Vehicle treatment (ethanol) was used as control. The induction of KO in MEFs was highly efficient, as indicated by PCR analysis of the XBP-1 locus in treated cell DNA samples (Fig. 2E). To explore whether the differences seen for the immortalized MEFs are directly related to the presence or absence of XBP-1, the growth of MCMV was measured in RERT/XBP-1^f/f^ MEFs treated 48 h with 4-OHT or vehicle prior to infection. Treatment with 4-OHT resulted in reduced growth in a similar MOI-dependent fashion as was observed for XBP-1 KO cells (Fig. 2A, MOI = 0.1 and Fig. 2B, MOI = 1). Experiments using the MCMV-GFP resulted in a 50% decrease in the number of GFP expressing cells at 24 hpi when XBP-1 deletion was induced (Fig. 2C). It should be noted that the level of GFP expressing cells varied between experiments, probably owing to differences in the behavior of the primary cells in the cultures. However, the advantage of XBP-1 proficient cells remained consistent over the KO. Because MEFs were prepared from mixed male and female embryos, it was important to validate that the reduction in GFP expression was not attributed to the effect of 4-OHT on the endogenous estrogen receptors. To address this issue MEFs of RERT/XBP-1^WT/WT^ mice prepared in the same manner were subjected to 4-OHT or vehicle treatment and infection with MCMV-GFP. In this strain, the XBP-1 gene is not floxed and is therefore not affected by the 4-OHT treatment. Indeed, PCR analysis of genomic DNA revealed no change in the XBP-1 locus following 4-OHT treatment (Fig. 2F) and the ratio of GFP-expressing cells in either vehicle or 4-OHT treated RERT/XBP-1^WT/WT^ fibroblasts, was similar (Fig. 2D).

To make sure that this effect was not restricted to GFP, viral protein levels of an immediate-early, an early, and a late protein were measured by immunoblotting. As expected, deletion of XBP-1 caused a reduction in tested viral protein expression as well (Fig. 2G). To verify that the reduction in viral protein expression is not related to reduction in global protein synthesis due to ER stress induction we pulse labeled the cells with ^35^S-methionine. We observed no obvious differences in the amount of newly synthesized proteins between mock or MCMV infected cells treated with vehicle compare to 4-OHT (Fig. 2H). The comparable levels of cellular protein synthesis indicate that XBP-1 is specifically required for optimal expression of viral genes.

### Deletion of XBP-1 at the immediate early phase of infection also impedes viral growth and delays MCMV protein expression

**Figure 3:**
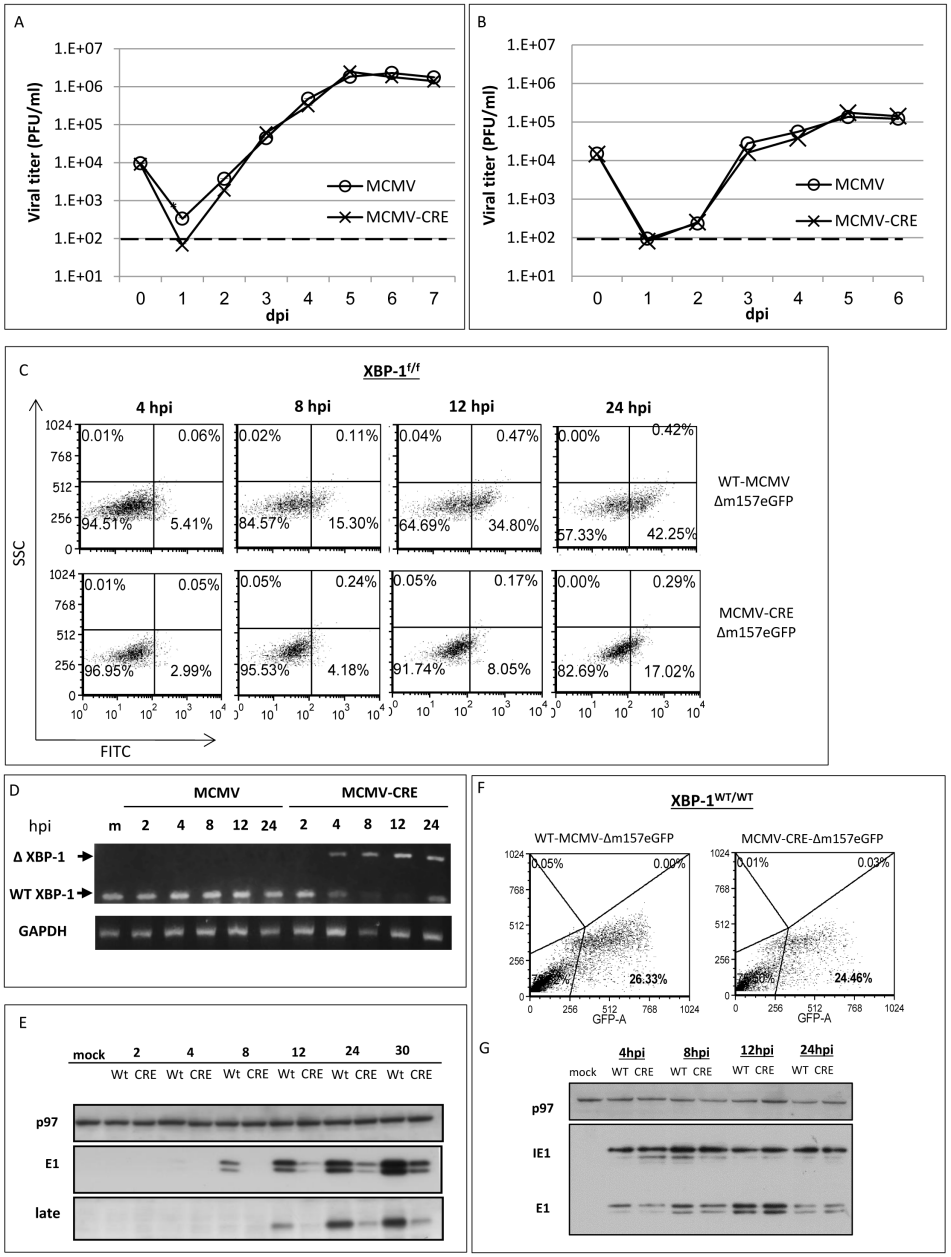
Induction of XBP-1 KO by virally-encoded CRE also restricts viral growth and delays viral protein expression in MEFs. (A) Primary MEFs derived from XBP-1^f/f^ (A) or XBP-1^WT/WT^ (B) mice were infected with MCMV or MCMV-CRE at MOI = 0.1. Culture supernatants were collected at indicated time points and titers were determined using standard plaque assay. Primary MEFs derived from XBP-1^f/f^ mice (C) or XBP-1^WT/WT^ (F) were infected with either WT-MCMV-Δm157-EGFP or MCMV-CRE-Δm157-EGFP at MOI = 1. The expression of virus-derived GFP was monitored using flow cytometry. GFP positive cells were gated and counted as infected cells. (D) The kinetics of XBP-1 deletion following infection with CRE expressing virus was demonstrated by semi-quantitative RT-PCR reaction. (The expression of intrinsic viral proteins in XBP-1^f/f^ (E) or XBP-1^WT/WT^ (G) infected MEFs was measured by Western blot analysis. Cellular p97 served as loading control. Data is representative from one experiment out of two (D, F,G) or three preformed (C, E). (A, B) Results are shown as mean ±SEM of at least three replicates. * P<0.05, ** P<0.01.

In both cellular systems described above, MEFs were deleted for XBP-1 irrespective of MCMV infection. Thus, its deletion may affect early steps of viral infection, such as the attachment or endocytosis, leading to the observed delay in viral protein synthesis. To gain better insight about the process affected by XBP-1, we induced the deletion of XBP-1 immediately after the entry phase, in the course of viral infection. To this end, we used MCMV viruses that express Cre recombinase to infect fibroblasts from XBP-1^f/f^ mice. In a manner consistent with the previous data, the infection with MCMV-CRE at an MOI of 0.1 yielded lower titers at 1 dpi than WT MCMV (Fig. 3A). Control experiments with WT MEFs, in which expression of Cre does not affect XBP-1, resulted in similar growth of both MCMV and MCMV-CRE (Fig. 3B). These data clearly demonstrate that the retardation in viral growth, although modest, is a result of a post-entry event and is not a consequence of reduced infection of cells lacking XBP-1.

To inspect the first infection cycle in these settings more carefully we cloned GFP into the genome of MCMV and MCMV-CRE in lieu of m157, a gene that does not affect viral growth in vitro. WT-MCMV-Δm157-eGFP or MCVM-CRE-Δm157-eGFP viruses were used to infect XBP-1^f/f^ fibroblasts and GFP expression was followed by flow cytometry. Similar to our previous observation, the number of GFP expressing XBP-1^f/f^ MEFs was reduced in cultures infected with MCMV-CRE-Δm157-eGFP compare to WT-MCMV-Δm157-eGFP (Fig. 3C). To validate that this reduction can be attributed to viral-induced deletion of XBP-1 we analyzed the XBP-1 locus throughout the infection and observed recombination as early as 4 hpi (Fig. 3D). As seen for the KO cell line and RERT/XBP-1^f/f^ MEFs treated with 4-OHT, the attenuation in viral protein synthesis was general, as assessed by Western blotting to the three representative viral proteins (Fig. 3E). The effect was directly related to the expression of XBP-1 since the kinetics of viral proteins expression was comparable when WT-MCMV-Δm157-eGFP and MCMV-CRE-Δm157-eGFP were used to infect WT MEFs (Fig. 3F, 3G).

### XBP-1 does not affect the accumulation of viral DNA in host nuclei, but is required for optimal transcription of viral genes

**Figure 4:**
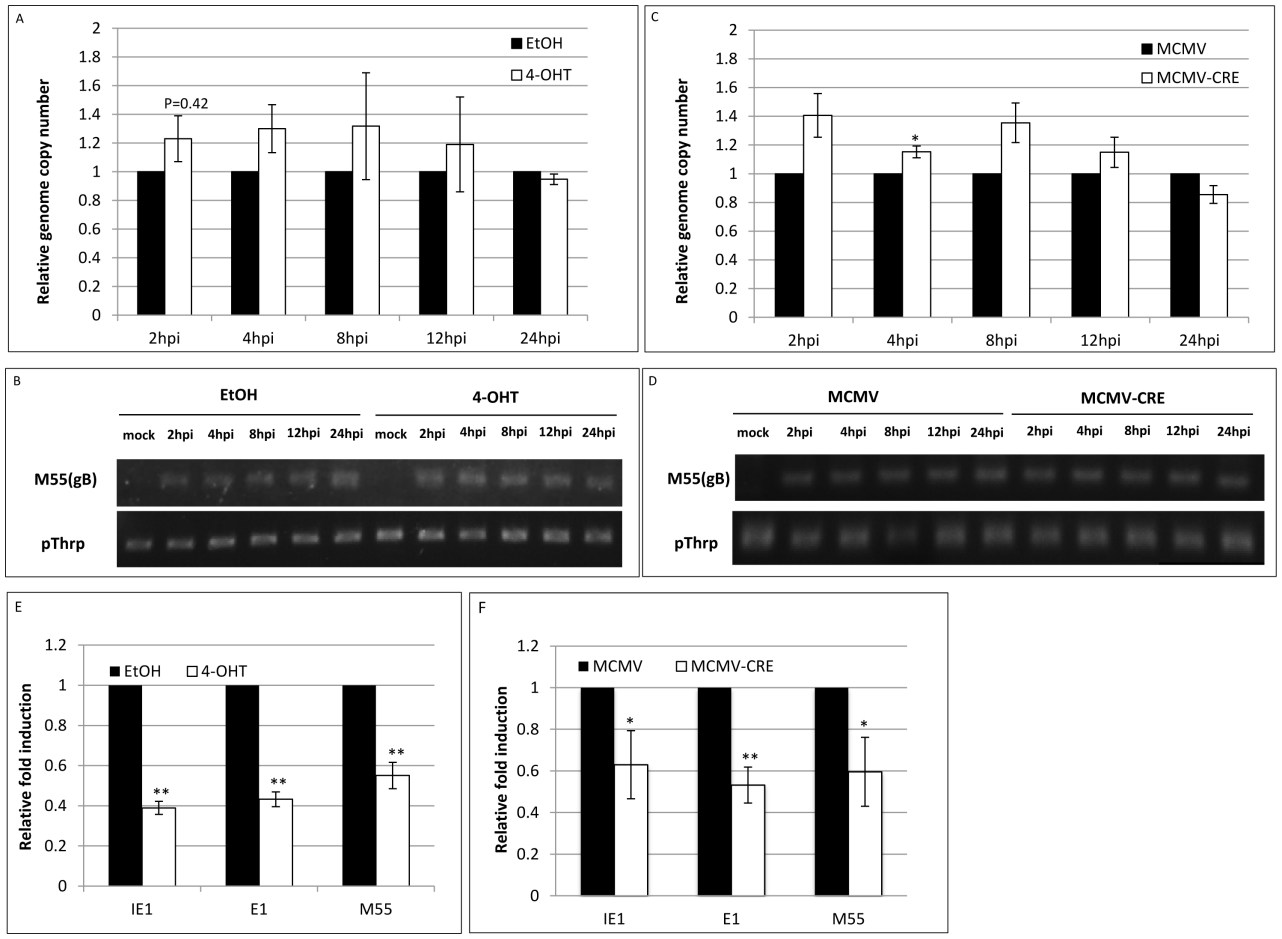
XBP-1 does not affect the accumulation of viral DNA in host nuclei, but is required for optimal transcription of viral genes. Primary MEFs derived from RERT/XBP-1^f/f^ mice were treated with vehicle (EtOH) or 4-OHT for 48 h and infected with MCMV-GFP at MO1 = 1. DNA was purified from nuclear extracts of infected cells at indicated time points. The number of MCMV genome copies in the nucleus was calculated from qPCR data (A). Samples were loaded on 2% agarose gel (B). Untreated RERT/XBP-1^f/f^ MEFs were also infected with MCMV or MCMV-CRE and genome copy number was analyzed the same way (C, D). The transcription levels of representative MCMV genes were analyzed by quantitative RT-PCR and were calculated relative to expression levels in EtOH treated cells at 12 hpi (E) or MCMV infected cells (F). Results are shown as mean ±SEM of two (A, C) or three (E, F) replicates. * P<0.05, ** P<0.01.

The experiments with MCMV-CRE provided conclusive evidence of a post-entry role of XBP-1 and encouraged us to inspect consequent events in early MCMV infection. Among its other activities, XBP-1 has been shown to link UPR and autophagy in neurons through a direct interaction between XBP-1u and FoxO1. In these cells the deletion of XBP-1 strongly enhanced autophagy [46]. This involvement of XBP-1 in autophagy may enhance the degradation of viral DNA that penetrate the cytoplasm or the redirection of viral particles to destruction, explaining the retarded phenotype in XBP-1 deficient cells compare to WT [47]. To test this hypothesis we compared the accumulation of viral DNA in host's nuclei. Whether we used 4-OHT or vehicle treated RERT/XBP-1^f/f^ (Fig. 4A and 4B) or XBP-1^f/f^ MEFs infected with MCMV and MCMV-CRE (Fig. 4C and 4D), the absence of XBP-1 did not affect the level of viral DNA recovered from the nucleus. This result indicates that the contribution of XBP-1 to the optimization of viral protein synthesis occurs after the viral DNA reaches the nucleus.

One fundamental process that follows the entrance of viral DNA to the nucleus is the transcription of viral genes. Therefore, we measured the mRNA levels of immediate early, early and late representative viral genes. Interestingly, we observed a reduction in all specified mRNA levels either in infected MEFs chemically induced for XBP-1 deletion (Fig. 4E) or when XBP-1 was removed by a viral encoded CRE (Fig. 4F). Hence, the elimination of XBP-1 inhibits the production of viral mRNA. Taken together, we conclude that XBP-1 optimizes viral gene transcription, leading to efficient protein expression and subsequent viral growth in fibroblasts.

### Induction of XBP-1 KO restricts viral protein expression in peritoneal macrophages

While fibroblasts are highly permissive to MCMV infection, their relevance for MCMV dissemination in vivo is limited. In vivo, MCMV also infects epithelial cells, endothelial cells and cells from the myeloid lineage, such as monocytes and macrophages [48]. Since macrophages play an important role in both viral dissemination and pathogenesis [49], we decided to explore the effect of XBP-1 KO in these cells. We focused on peritoneal exudate macrophages (PMs), extracted from mice challenged with a thioglycolate broth. This challenge induces a massive infiltration of circulating macrophages into the peritoneal cavity. To confirm macrophage identity we stained the extracted cells for F4/80 and analyzed them by flow cytometry. More than 95% of cells in culture expressed high levels of this marker (not shown).

**Figure 5:**
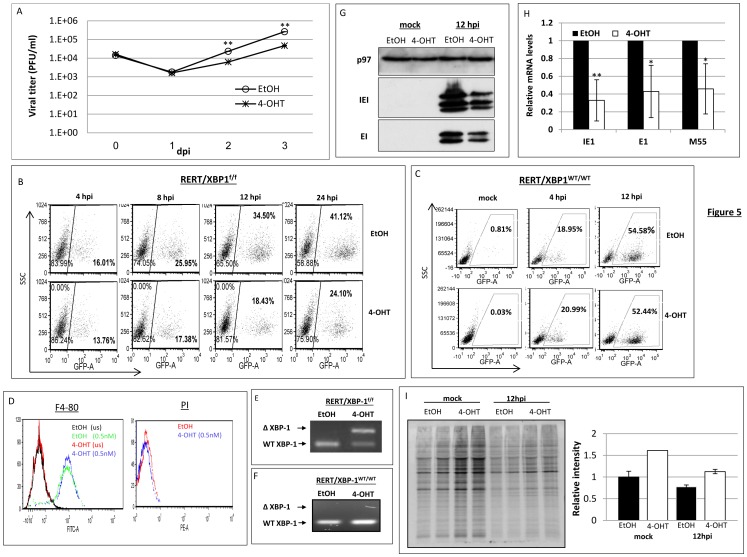
Induction of XBP-1 deletion prior to MCMV infection decelerates viral protein expression in peritoneal macrophages (PMs). Primary PMs were isolated from RERT/XBP-1^f/f^ mice 4 days after i.p. injection of thioglycolate and treated with vehicle (EtOH) or 4-OHT for 48 h. Cells were than infected with MCMV-GFP at MO1 = 0.5. Culture supernatants were collected at indicated time points and titers were determined using standard plaque assay (A). The expression of virus-derived GFP in RERT/XBP-1^f/f^ (B) and RERT/XBP-1^WT/WT^ (C) was monitored using flow cytometry. Cells were treated with either EtOH or 4-OHT for 48 h prior to infection with MCMV-GFP at MOI = 1 and GFP positive cells were gated and counted as infected cells. (D) FACS analysis of cells following staining with propidium iodide or F4/80 Ab. (E, F) PCR analysis of the genomic deletion of XBP-1 following EtOH or 4-OHT treatment. Upper band represents deleted allele. (G) Expression of intrinsic viral proteins at 12 hpi was measured by Western blot analysis. Cellular p97 served as loading control. (H) The transcription levels of representative MCMV genes were analyzed by quantitative RT-PCR and were calculated relative to expression levels in EtOH treated cells at 12 hpi. (I) Total protein synthesis was assessed by radioactive pulse labeling and quantified using ImageJ. Data is from one representative experiment out of two (C, D, I) or three performed (B, G, H). Results are shown as mean ±SEM of three replicates. * P<0.05, ** P<0.01.

To obtain XBP-1 deficient PMs, we utilized the inducible KO system again. PMs were isolated from RERT/XBP-1^f/f^ mice and treated for 48 h with either vehicle or 4-OHT. Importantly, the removal of XBP-1 by 4-OHT treatment compared to vehicle had no effect on macrophage viability and identity, as indicated by propidium iodide (PI) staining and F4/80 respectively (Fig. 5D). We optimized a protocol to assess viral propagation in PMs. Because viability of PMs is severely compromised 5 to 6 days after isolation and because 48 h treatment with 4-OHT was required to efficiently induce the deletion of XBP-1(Fig. 5E), growth analysis was restricted to 3 dpi. Since macrophages are less permissive to MCMV infection than fibroblasts, we used an MOI of 0.5 over 0.1. Notably, progeny titers in 1 dpi were similar but viral growth was significantly retarded in the XBP-1 KO macrophages at 2 and 3 dpi (Fig. 5A). This result imply that despite a possible difference in the kinetics of MCMV infection in macrophages compare to fibroblasts, XBP-1 plays a general role in supporting MCMV growth at the cellular level.

**Figure 6:**
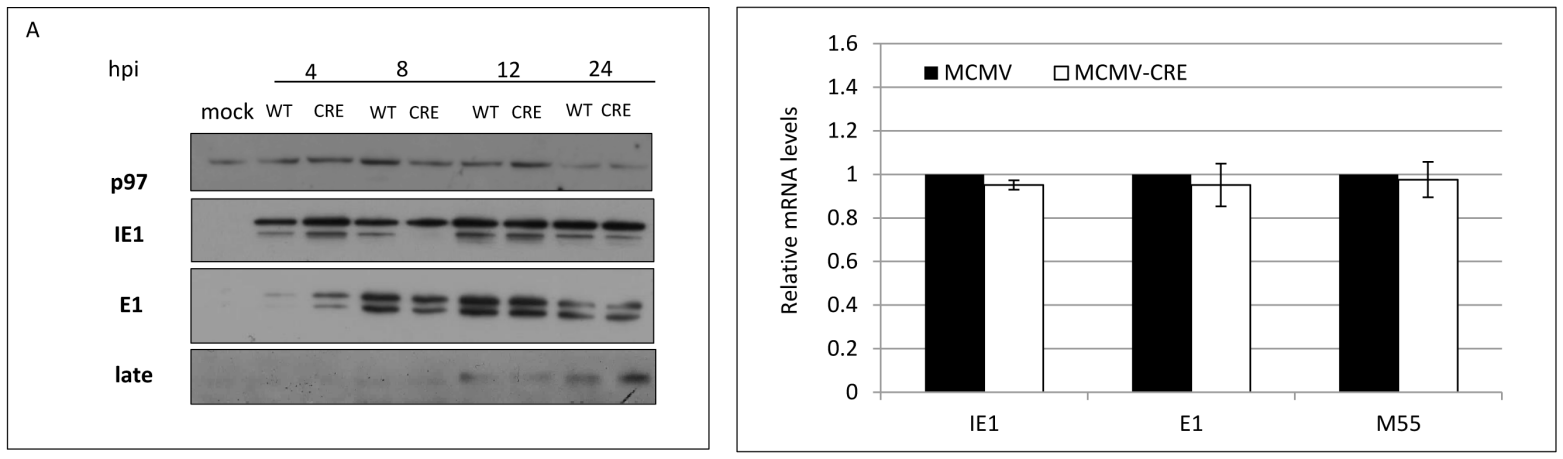
Viral infection-induced deletion of XBP-1 does not affect viral RNA and protein expression in PMs. Primary PMs derived from XBP-1^f/f^ mice were infected with MCMV or MCMV-CRE at MOI = 1 and the expression of intrinsic viral proteins (A) was measured by Western blot. Cellular p97 served as loading control. (B) The transcription levels of representative MCMV genes were analyzed by quantitative RT-PCR at 12 hpi and calculated relative to expression levels in mock infected cells. (A) Data is from one representative experiment out of three performed. (B) Results are shown as mean ±SEM of three replicates.

To inspect the first infection cycle we utilized the GFP expressing virus. However, to obtain detectable GFP levels and efficient viral mRNA recovery, we infected cells with an MOI of 1.5 and added a centrifugal enhancement. In accordance with data obtained from fibroblasts, the proportion of GFP-expressing cells was about 2-fold lower in PMs induced for XBP-1 deletion compared to WT (Fig. 5B). Treatment with 4-OHT did not affect the kinetics of GFP expression in infected PMs derived from RERT/XBP-1^WT/WT^ mice, in which the XBP-1 locus remained intact (Fig. 5C and 5F). Protein levels and their corresponding mRNA levels of IE1 and E1 at 12 hpi were also lower in induced cells (Fig. 5G and 5H). This occurred without significantly affecting total protein synthesis, as assessed by metabolic labeling (Fig. 5I). We conclude that, as in fibroblast, preemptive deletion of XBP-1 in peritoneal macrophages decelerates the expression of various viral genes primarily by reducing transcription. Interestingly, when we infected XBP-1^f/f^ PMs with MCMV or MCMV-CRE, we observed no differences. Deletion of XBP-1 from PMs upon infection with the CRE expressing virus did not alter the expression of neither viral mRNA nor protein (Fig. 6A, 6B). Hence, in PMs the preemptive deletion of XBP-1 interferes with early stages of viral infection, but when deletion is induced by the virus itself it has no effect, at least under these experimental conditions.

### Preemptive ubiquitous deletion of XBP-1 attenuates MCMV infectivity in vivo

**Figure 7:**
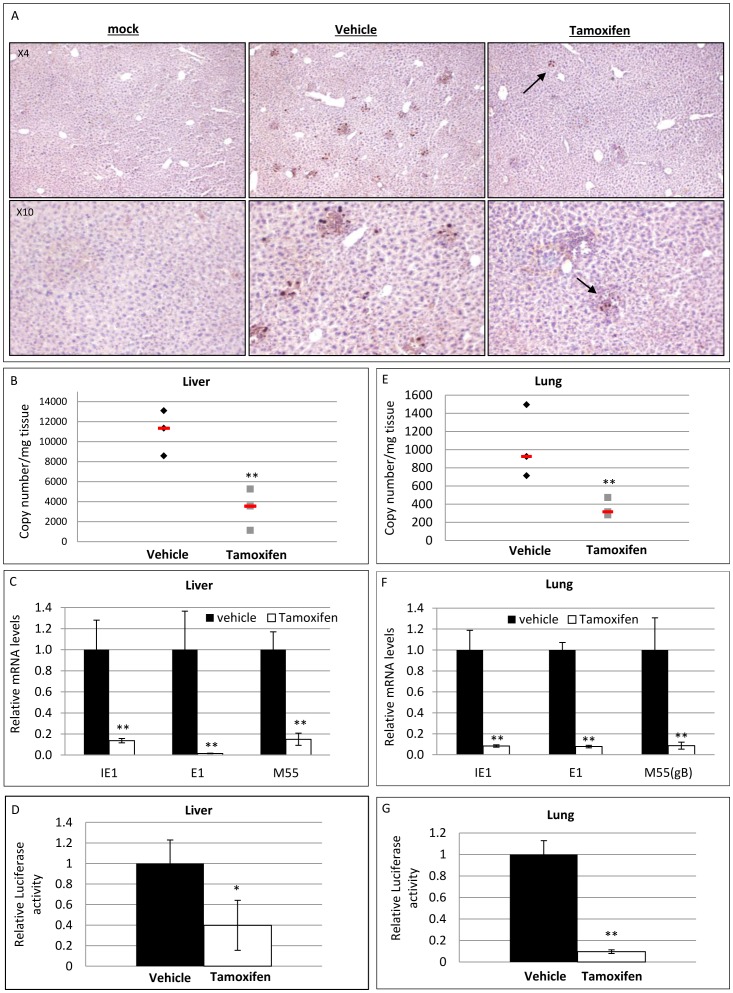
Deletion of XBP-1 impairs MCMV infection in vivo. RERT XBP-1^f/f^ mice were treated with either vehicle or tamoxifen. One week later mice were infected with a luciferase expressing MCMV. Mice were sacrificed 4 dpi and organs were extracted. (A) Liver paraffin-embedded sections were decorated with αIE1 to visualize viral foci. (B+E) viral genome copy number in mg tissue was determined using quantitative RT-PCR analysis of viral M55(gB) and the host gene pThrp in liver (B) and lung (E) (n = 3). The expression of representative viral RNA (IE1, E1 and M55(gB)) in liver (C) or lung (F) was measured by quantitative RT-PCR and calculated relative to vehicle treated animals (n = 6 (vehicle), n = 8 (tamoxifen)). Luciferase activity, representing viral protein expression was measured in liver (D) or lung (G) (n = 6 (vehicle), n = 7 (tamoxifen). Results are shown as mean ± SEM and are combined from two independent experiments. * P<0.05, ** P<0.01.

The data depicted above implied that the requirement of XBP-1 for MCMV infection may be cell type-dependent. This prompted us to explore the overall effect of XBP-1 KO on MCMV acute infection in vivo. For this purpose we induced the ubiquitous deletion of XBP-1 in RERT/XBP-1^f/f^ male mice using a series of three subcutaneous injection of tamoxifen. A week after the last injection, mice were infected i.v. with 2×10^5^ PFU of BAC-derived MCMV that expresses the firefly luciferase gene (Luc-MCMV). The high sensitivity of the luminescence assay allows for detection of scarce viral-encoded proteins in isolated tissue, providing a major advantage in cases of low productivity. To evaluate the extent of acute infection, mice were sacrificed 4 days postinfection and organs were harvested. Since hepatocytes are targets of primary infection, livers were analyzed for presence of virus-encoded molecules. We observed statistically significant decreases in all infection parameters tested in the tamoxifen induced animals compared to WT. The number of foci of infected cells positive for intra-nuclear staining of the IE1 protein was lower in liver sections of tamoxifen-treated mice liver sections (Fig. 7A). In accordance with decreased IE1 expression, the luciferase activity levels in liver homogenates of induced mice were 2.5-fold lower than control (Fig. 7D). Moreover, MCMV genome copy number was decreased in the liver of tamoxifen treated mice (Fig. 7B) in a manner consistent with a reduced mRNA levels of IE1, E1 and M55/gB as assessed by qPCR (Fig. 7C). Similar differences in genome copy number (Fig. 7E), transcription (Fig. 7F) and luciferase activity (Fig. 7G) were obtained for the lungs of infected animals. Although viral DNA, RNA and proteins were detected in the tissues, infective viral progeny could not be recovered. Overall we were able to demonstrate that systemic deletion of XBP-1 prior to infection attenuates MCMV ability to propagate in vivo during acute infection.

### Post-entry deletion of XBP-1 attenuates MCMV infectivity in vivo

**Figure 8:**
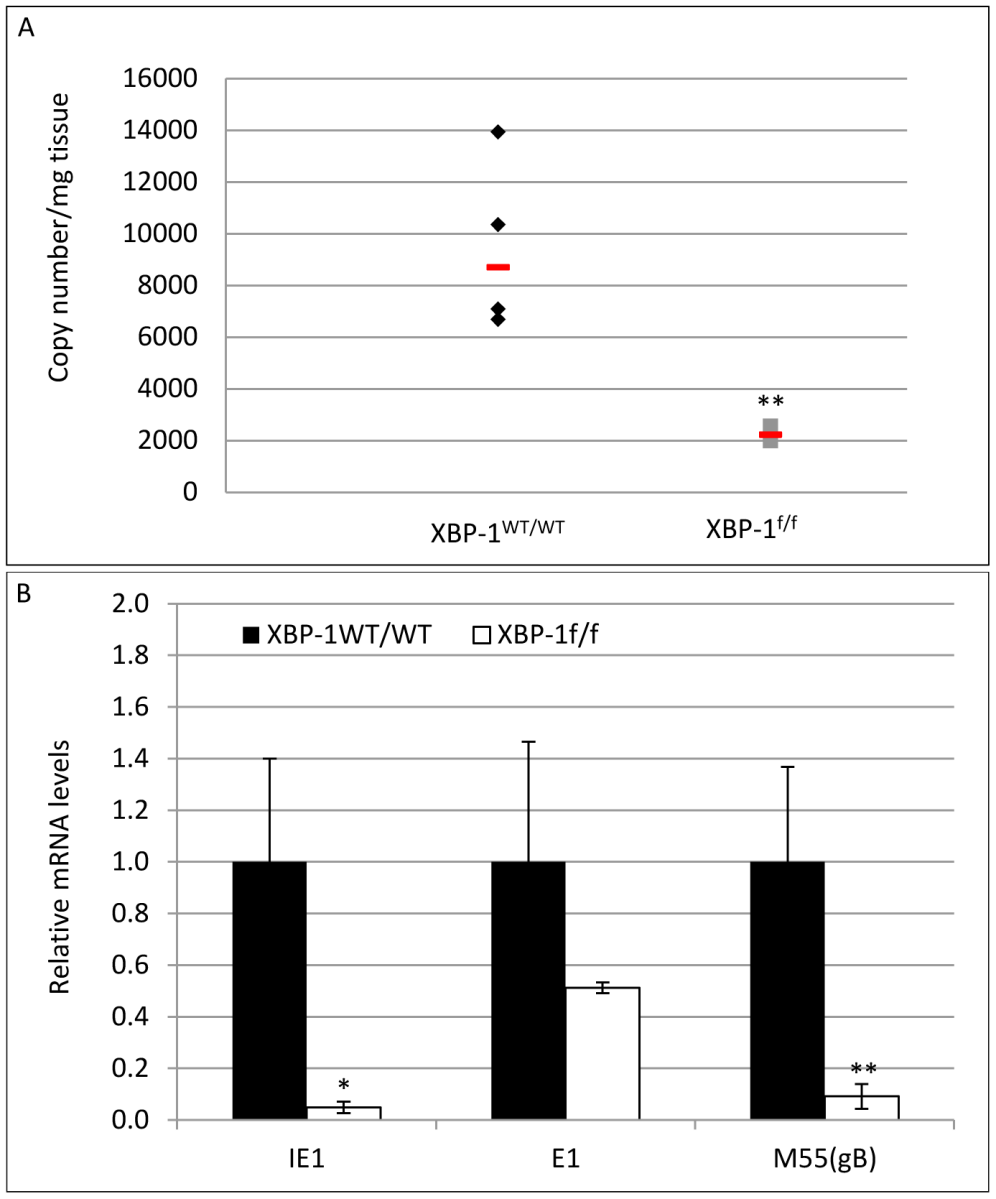
Viral induced deletion of XBP-1 impairs viral DNA replication and gene expression in vivo. XBP-1^WT/WT^ and XBP-1^f/f^ mice infected with 10∧5 PFU of MCMV-CRE. Mice were sacrificed 4 dpi and livers were extracted. (A) Viral genome copy number in mg tissue was determined using quantitative RT-PCR analysis of viral M55(gB) and the host gene pThrp (n = 3 (XBP-1^f/f)^, n = 4 (XBP-1^WT/WT^)). The expression of representative viral RNAs (IE1, E1 and M55(gB)) in liver was measured by RT-PCR analysis and calculated relative to WT animals. Results are shown as mean ±SEM. * P<0.05, ** P<0.01.

**Figure 9:**
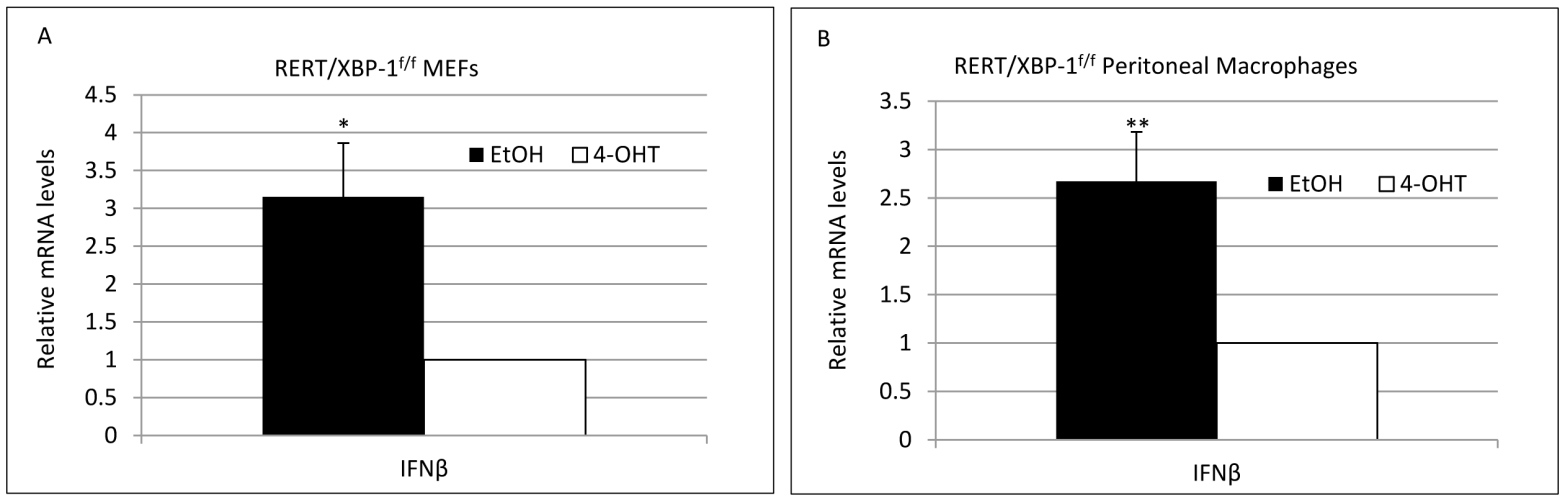
IFNβ mRNA levels are lower following MCMV infection in XBP-1 deleted cells. RERT/XBP-1^f/f^ MEFs (A) and PMs (B) were treated with EtOH or 4-OHT for 48 h. Cells were then infected with MCMV at MOI = 1 and the mRNA levels of IFN-β were measured by quantitative RT-PCR at 12 hpi. Fold induction was calculated relative to expression levels in mock infected cells. (B) Results are shown as mean ±SEM of three replicates * P<0.05, ** P<0.01.

In the RERT model XBP-1 was deleted ubiquitously and irrespectively of MCMV infection. We were also interested to explore whether viral-induced removal of XBP-1 after cell entry also leads to attenuation of viral mRNA and protein expression. To this end we infected XBP-1^f/f^ or XBP-1^WT/WT^ mice with MCMV-CRE. A 4-fold lower viral genome copy number was measured in the XBP-1^f/f^ liver homogenates compared to XBP-1^WT/WT^ and a significant decrease in IE1, E1 and M55/gB transcription levels was measured by qPCR (Fig. 8A and 8B). These data suggests that XBP-1 is intrinsically required for optimal viral growth under an in vivo setting, and its loss upon infection hinders MCMV gene expression and DNA replication. This finding is somewhat surprising when considering the involvement of XBP-1 in regulation of the IFNβ-mediated antiviral response. It has been shown that ER stress conditions synergistically increase IFNβ expression and secretion and that XBP-1 association with CBP/p300 mediates this promoter enhancement [24]. Although this was shown in the context of toll-like receptor engagement and not under normal conditions of infection, we tested IFNβ transcription levels in our models. Indeed, we saw that the induction of IFNβ expression following MCMV infection was higher in WT cells than in those induced for XBP-1 deletion (Fig. 9A and 9B). This was also the case in tissues isolated from in-vivo infection experiments (not shown). This led us to assume that apart from its antiviral activity, XBP-1 might possess a pro-viral role that allows the promotion of viral genes transcription, conferring a lower infection rate in the absence of XBP-1 despite lower levels of IFNβ.

## Discussion

The UPR in the context of viral infection can serve the pathogen by improving the survival of host cells under enhanced protein load in the ER and by ensuring the fidelity of viral glycoprotein synthesis and folding. On the other hand, the UPR may serve to the advantage of the host by promoting the production of antiviral cytokines such as interferons and proinflammatory responses, such as the NF-ĸb pathway [50], and directing the infected cells to apoptosis through the ER stress pathway. The discovery that MCMV targets IRE1, the most conserved and central element of the UPR [33], strongly implies that the UPR serves better the host and thus MCMV may tolerate the compromise in ER functions for the sake of other activities governed by IRE1.

The nuclease activity of IRE1 is not restricted to the mRNA of XBP-1. While XBP-1 mRNA undergoes splicing by IRE1, other RNA molecules are subjected to degradation through RIDD. This degradation can generate small RNA molecules that activate the RIG-I sensor and downstream proinflammatory signaling cascades [51]. RIDD also has controversial roles in promoting ER stress-mediated apoptosis, probably depending on the cell type and the exact experimental conditions. Inhibitors of IRE1 were demonstrated to promote cell death in B cell malignancies [52], while preserving viability of β islet cells [53]. IRE1, through RIDD, promoted the proapoptotic activity of caspase 2 [19], while negating apoptosis through the degradation of death receptor 5 mRNA [54]. All of these make it difficult to predict the exact roles of IRE1 in the context of dynamic and complex processes, such as viral infection.

Common to all RIDD activities is their enhancement by the deletion of XBP-1 [55], [56]. In this study we rigorously addressed the role of XBP-1 in MCMV infection. Two extreme scenarios can be postulated. Removal of XBP-1 could benefit the virus, indicating that the virus aims at degrading IRE1 for its XBP-1 splicing activity. In contrast, removal of XBP-1 could harm the virus, suggesting that the virus by degrading IRE1 “sacrifices” XBP-1 benefits, for eliminating the more severe toxic outcomes of RIDD. Using three independent approaches to study the role of XBP-1 in the course of MCMV infection in fibroblasts, it was clear that removal of XBP-1 either before infection or early after it, delays the expression of viral proteins (Fig. 1–3 and 5). The inhibition in viral protein synthesis appeared to attenuate synchronously all kinetic classes of viral proteins, as a similar pattern of expression was observed for immediate early, early, and late proteins including the virally encoded GFP reporter (Fig. 1–3 and 5). Importantly, the reduction in viral protein expression was not a consequence of global attenuation in protein synthesis, suggesting that the engagement of PERK was efficiently counteracted in fibroblasts and macrophages (Fig. 1F, 2H, 5I). Previous studies showed that protein synthesis was not affected also in the context of HCMV infection [9]. Interestingly, the reduction in viral protein synthesis was correlated with reduced viral mRNA levels (Fig. 4E, 4F, 5H). Moreover, mRNA level of viral proteins were reduced to a similar extent for all mRNA tested, suggesting that XBP-1 is required for a yet unknown fundamental early event that affects viral gene transcription and the initiation of viral DNA replication.

Early events of viral infection include cell entry, transport of the capsids to the cell nucleus, uncoating, injection of the genome into the nucleus and the actual initiation of gene expression and viral DNA replication. Experiments with Cre recombinase expressing viruses drove the conclusion that XBP-1 is not affecting cell entry, as its removal confers viral disadvantage even when occurring only after the virus enters the cell and the Cre protein is being expressed (Fig. 3). In accordance with that, a similar kinetics of viral DNA entry into the nucleus of WT and XBP-1 KO cells was demonstrated (Fig. 4A–4D), suggesting no role for XBP-1 in trafficking of the capsid or uncoating. The attenuated levels of virally encoded mRNA suggest that XBP-1 can optimize viral gene transcription, directly or indirectly. The large enhancer region located upstream of major immediate early (MIE) locus was shown to be occupied by various transcription factors that regulate transcription of the MIE genes [57]. It is tempting to entertain the possibility that a potent transcription factor as XBP-1 is recruited by MCMV to enhance viral transcription. This possibility is even more appealing considering the fact that XBP-1 activates the transcription of critical viral genes in other herpes viruses, for instance of the EBV-encoded LMP1 or the KSHV RTA [58], [59]. However, analysis of XBP-1 binding to MCMV genome has not been performed yet. The fact that an increased MOI alleviates the contribution of XBP-1 to viral propagation can support this idea, if in higher dosage virus can reach optimal propagation without XBP-1. In a manner analogous to XBP-1, lack of ATF4 impairs MCMV infection at low MOI, but is completely dispensable for viral growth at high MOI. However, in contrast to XBP-1, analysis of MCMV progression in ATF4 deficient hosts indicated a defect at late times p.i., a time that probably corresponds to the second round of infection [31]. In our hands the role of XBP-1 was confined to the first two days at MOI of 0.1. It should be noted that we have not analyzed MOI of 0.01 as was done for ATF4. It is likely that at much lower MOI the differences between XBP-1 deficient and proficient cells will increase in a manner more similar to ATF4. Regardless of the experimental details, our study strengthens the hypothesis that ER homeostasis plays a role for MCMV progression, but is not essential.

Macrophages and fibroblasts exert different transcriptional programs following MCMV infection. For example, IFN-γ was demonstrated to inhibit the growth of MCMV in macrophages but not in fibroblasts [60]. In addition a set of viral genes are specifically expressed in macrophages and not in other cell types [61]. We therefore decided to test specifically the requirement of XBP-1 in macrophages. *A priori* deletion of XBP-1 resulted in a similar retardation of infection as seen in fibroblasts (Fig. 5A, 5B, 5G and 5H). However, when using Cre-expressing viruses, no effect was seen for lack of XBP-1 (Fig. 6A and 6B). Interestingly, in some experiments we even recorded an accelerated propagation of the Cre virus in XBP-1^f/f^ macrophages. This effect can be due to the mixture of functions governed by XBP-1, related in addition to its effect on ER physiology also to its role in modulating the interferon response and other signaling pathways, more relevant for immune cells. Indeed, measurements of the IFNβ levels were reduced in the XBP-1 KO cells (Fig. 9). While this may have limited importance to viral propagation in fibroblasts, it may promote the infection in macrophages, leading to robust induction of the anti-viral state thus allowing better viral clearance.

Further support for the role of XBP-1 in MCMV infection comes from the in vivo studies. We used two approaches. In one we ubiquitously removed XBP-1 before infection and in the second we used Cre-expressing viruses to remove XBP-1 only in infected cells, an approach that was previously used to demonstrate the role of Blimp1 for MHV-68 infection [62] and to monitor viral dissemination in vivo [63]. Both models yielded a similar effect for XBP-1 deletion. The effect was more robust than what was measured for fibroblasts, indicating that in vivo, XBP-1 probably plays a more central role for MCMV acute infection (Fig. 7 and 8). The importance of XBP-1 at low MOI and its pronounced effect in vivo suggest that under MCMV reactivation from latency, conditions in which viral dosage is minimal, XBP-1 should display an even larger significance.

In conclusion, our study reveals the multifaceted importance of the IRE1/XBP-1 pathway for MCMV infection. It is likely that the manipulation of this pathway by cytomegaloviruses is related to the immunological roles of the UPR rather than to its role in ER quality control. Our data show that while at the early stages of infection IRE1 is important by executing XBP-1 splicing, at later times when the cells are committed to viral protein synthesis and generation of viral progeny, IRE1 becomes toxic and the virus targets it for degradation [33]. The exact activities of IRE1 that MCMV and HCMV evolved to avoid at the late phases of infection remain to be elucidated. Understanding the spatial and cellular specific roles of the UPR in the context of infection may allow the design of therapies that may be effective also in immunocompromised patients, severely affected by this pathogen.
